# Lung ultrasound in the critically ill

**DOI:** 10.1186/2110-5820-4-1

**Published:** 2014-01-09

**Authors:** Daniel A Lichtenstein

**Affiliations:** 1 Service de Réanimation Médicale, Hôpital Ambroise-Paré, University Paris-West, Boulogne, France

**Keywords:** Lung ultrasound, Acute respiratory failure, Acute circulatory failure, Pulmonary oedema, Pulmonary embolism, Pneumonia, Pneumothorax, Interstitial syndrome, Fluid therapy, Haemodynamic assessment, Intensive care unit

## Abstract

Lung ultrasound is a basic application of critical ultrasound, defined as a loop associating urgent diagnoses with immediate therapeutic decisions. It requires the mastery of ten signs: the bat sign (pleural line), lung sliding (yielding seashore sign), the A-line (horizontal artifact), the quad sign, and sinusoid sign indicating pleural effusion, the fractal, and tissue-like sign indicating lung consolidation, the B-line, and lung rockets indicating interstitial syndrome, abolished lung sliding with the stratosphere sign suggesting pneumothorax, and the lung point indicating pneumothorax. Two more signs, the lung pulse and the dynamic air bronchogram, are used to distinguish atelectasis from pneumonia. All of these disorders were assessed using CT as the “gold standard” with sensitivity and specificity ranging from 90% to 100%, allowing ultrasound to be considered as a reasonable bedside “gold standard” in the critically ill. The BLUE-protocol is a fast protocol (<3 minutes), which allows diagnosis of acute respiratory failure. It includes a venous analysis done in appropriate cases. Pulmonary edema, pulmonary embolism, pneumonia, chronic obstructive pulmonary disease, asthma, and pneumothorax yield specific profiles. Pulmonary edema, e.g., yields anterior lung rockets associated with lung sliding, making the “B-profile.” The FALLS-protocol adapts the BLUE-protocol to acute circulatory failure. It makes sequential search for obstructive, cardiogenic, hypovolemic, and distributive shock using simple real-time echocardiography (right ventricle dilatation, pericardial effusion), then lung ultrasound for assessing a direct parameter of clinical volemia: the apparition of B-lines, schematically, is considered as the endpoint for fluid therapy. Other aims of lung ultrasound are decreasing medical irradiation: the LUCIFLR program (most CTs in ARDS or trauma can be postponed), a use in traumatology, intensive care unit, neonates (the signs are the same than in adults), many disciplines (pulmonology, cardiology…), austere countries, and a help in any procedure (thoracentesis). A 1992, cost-effective gray-scale unit, without Doppler, and a microconvex probe are efficient. Lung ultrasound is a holistic discipline for many reasons (e.g., one probe, perfect for the lung, is able to scan the whole-body). Its integration can provide a new definition of priorities. The BLUE-protocol and FALLS-protocol allow simplification of expert echocardiography, a clear advantage when correct cardiac windows are missing.

## Lung ultrasound in the critically ill

The possibility of exploring the lung using ultrasound, at the bedside and noninvasively, is gaining popularity among intensivists. Lung ultrasound would be of minor interest if the usual tools (bedside radiography, CT) did not have drawbacks (irradiation, low information content for radiography, need for transportation…). This review will show that ultrasound can be used instead of CT in many cases.

We used ultrasound first in 1983, on occasion in François Fraisse’s ICU in 1985–1989, then since 1989 in François Jardin’s ICU, using the on-site 1982 ADR-4000 devoted to cardiac assessment, in actual fact suitable for whole body and lung assessment and not larger than nowadays laptops [1]. At this time, although an old idea [2], ultrasound was not routine in the ICUs and had neglected this vital organ [3]. Many doctors thought that lung ultrasound was unfeasible [4,5]. For demonstrating that this dogma was wrong, deciphering the artifact code was the easy part, but publishing was the hard one, far from finished. We will briefly consider the elements of this code, then major clinical uses.

Lung ultrasound is part of critical ultrasound, defined as a whole-body approach using simple machines, one universal probe, new applications [6,7]. Our priority was to publish lung ultrasound, leaving little time for developing basic fields (search for blood in trauma, venous line insertion…).

### Seven principles of lung ultrasound

1) Lung (and critical) ultrasound is performed at best using simple equipment.

2) In the thorax, gas and fluids have opposite locations, or are mingled by pathologic processes, generating artifacts.

3) The lung is the most voluminous organ. Standardized areas can be defined [8].

4) All signs arise from the pleural line.

5) Static signs are mainly artifactual [9,10].

6) The lung is a vital organ. The signs arising from the pleural line are foremost dynamic.

7) Almost all acute life-threatening disorders abut the pleural line, explaining the potential of lung ultrasound.

### Ten signs

**Figure 1:**
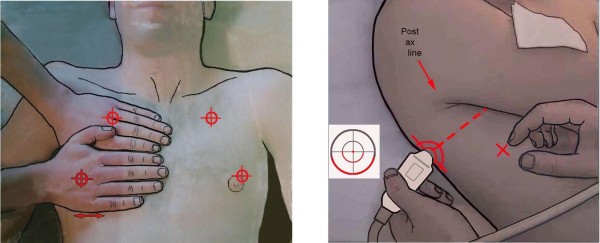
**Areas of investigation and the BLUE-points.** Two hands placed this way (size equivalent to the patient’s hands, upper hand touching the clavicle, thumbs excluded) correspond to the location of the lung, and allow three standardized points to be defined. The upper-BLUE-point is at the middle of the upper hand. The lower-BLUE-point is at the middle of the lower palm. The PLAPS-point is defined by the intersection of: a horizontal line at the level of the lower BLUE-point; a vertical line at the posterior axillary line. Small probes, such as this Japanese microconvex one (1992), allow positioning posterior to this line as far as possible in supine patients, providing more sensitive detection of posterolateral alveolar or pleural syndromes (PLAPS). The diaphragm is usually at the lower end of the lower hand. Extract from “Whole body ultrasonography in the critically ill” (2010 Ed, Chapter 14), with kind permission of Springer Science.

The Japanese microconvex probe we use is directly applied to the intercostal space. In the BLUE-protocol, three standardized points are the upper BLUE-point, lower BLUE-point and PLAPS-point [8] (Figure 1). In ARDS (Pink-protocol), a more comprehensive analysis includes four stages of investigation (anterior, lateral, posterior, apical). Ten signs are currently assessed. All our studies directly compared ultrasound with CT.

**Figure 2:**
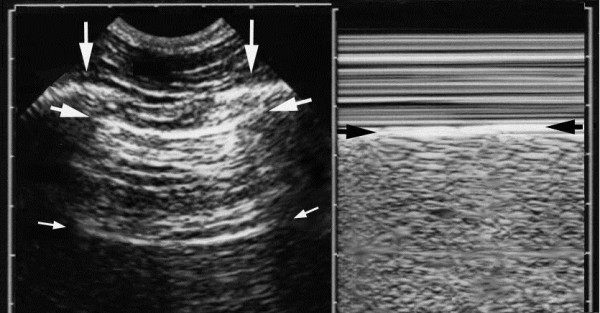
**Normal lung surface.** Left: Scan of the intercostal space. The ribs (vertical arrows). Rib shadows are displayed below. The pleural line (upper, horizontal arrows), a horizontal hyperechoic line, half a centimeter below the rib line in adults. The proportions are the same in neonates. The association of ribs and pleural line make a solid landmark called the bat sign. The pleural line indicates the parietal pleura in all cases. Below the pleural line, this horizontal repetition artifact of the pleural line has been called the A-line (lower, small horizontal arrows). The A-line indicates that air (gas more precisely) is the component visible below the pleural line. Right: M-mode reveals the seashore sign, which indicates that the lung moves at the chest wall. The seashore sign therefore indicates that the pleural line also is the visceral pleura. Above the pleural line, the motionless chest wall displays a stratified pattern. Below the pleural line, the dynamics of lung sliding show this sandy pattern. Note that both images are strictly aligned, of importance in critical settings. Both images, i.e., lung sliding plus A-lines make the A-profile (when found at the anterior chest wall). They give basic information on the level of capillary pressure. Extract from “Whole body ultrasonography in the critically ill” (2010 Ed, Chapter 14), with kind permission of Springer Science.

The pleural line generates the bat sign, a permanent landmark visible in all circumstances (agitated, bariatric patients, subcutaneous emphysema…). It indicates the parietal pleura (Figure 2).

The normal lung surface (Figure 2) associates lung sliding with horizontal repetitions of the pleural line, called A-lines. They indicate gas (physiological or free). Lung sliding is a to-and-fro movement at the pleural line, spreading below. The M-mode helps to understand that this movement is relative to superficial tissues (seashore sign). Lung-sliding indicates that the pleural line also contains the visceral pleura. Lung-sliding, physiologically more discrete at the upper parts, can be very discrete in pathological conditions. Some filters, especially average, dynamic noise, can make discrete lung-sliding more difficult to distinguish. We usually bypass all filters.

**Figure 3:**
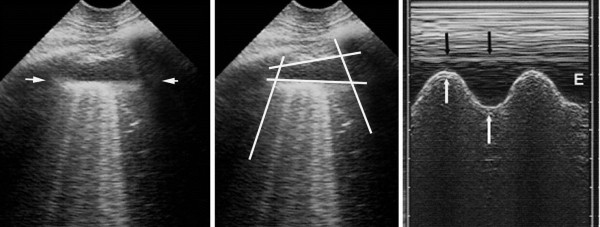
**Pleural effusion.** Left and middle: minute pleural effusion at the PLAPS-point. Below the pleural line, a line regular and roughly parallel to the pleural line can be seen: the lung line, indicating the visceral pleura (arrows). This line, together with the pleural line and the shadow of the ribs, display a kind of quad: the quad sign. Right: M-mode shows a movement of the lung line (white arrows) toward the pleural line (black arrows) on inspiration—the sinusoid sign, indicating also a free pleural effusion, and a viscosity enabling the use of small caliper needle if thoracentesis is envisaged. E, expiration. Quantitative data: this effusion found at the PLAPS-point has an expiratory thickness of roughly 13 mm, i.e., an expectedly small volume (study in progress). A 15-mm distance is our minimum required for safe diagnostic or therapeutic puncture, allowing to simplify the problem of modeling the real volume of an effusion (Ref. 14). Extract from “Whole body ultrasonography in the critically ill” (2010 Ed, Chapter 15), with kind permission of Springer Science.

Pleural effusion, a familiar field [1,11], became of interest to intensivists only recently. Our short probe is applied at the PLAPS-point, a posterior area accessible in supine patients, locating all free effusions, regardless their volume [8]. This direct approach generates standardized signs: the quad and sinusoid sign. The deep boundary of the collection is regular, roughly parallel to the pleural line, and is called the lung line (visceral pleura). This draws the quad sign (Figure 3). The lung-line moves toward the pleural line on inspiration. This draws the sinusoid sign, which also indicates a low viscosity, allowing fine needle insertion if needed (Figure 3). Our definition makes independent of the effusion color, traditionally anechoic: the most severe cases are echoic: empyema, hemothorax. For pleural effusions, sensitivity is 93%, specificity 97% [12,13]. Safe fluid withdrawal is possible even in radio-occult effusions in ventilated patients [12]. Small effusions can be withdrawn for diagnostic purpose (even if they appear smaller on CT), provided a 15-mm inspiratory distance is respected [12]. This safety distance allows fluid withdrawal without precise volume assessments, yet rough assessment is possible [14]. We don’t use ultrasound during thoracentesis.

**Figure 4:**
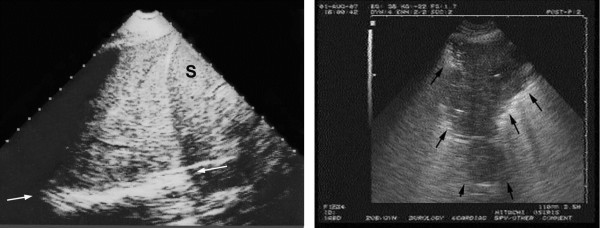
**Lung consolidation.** Two signs of lung consolidation. Left: a massive consolidation (probe at the PLAPS-point) invades the whole left lower lobe. No aerated lung tissue is present, and no fractal sign can be generated. The deep border is at the mediastinal line (arrows). The pattern is tissue-like, similar to the spleen (S). The thickness of this image is roughly 10 cm, a value incompatible with a pleural effusion. Image acquired using an ADR-4000 and a sectorial probe (1982 mobile technology) Right: a middle lobe consolidation, which does not invade the whole lobe. This generates a shredded, fractal boundary between the consolidation and the underlying aerated lung (arrows): the quite specific shred (or fractal) sign. Such an anterior consolidation generates the C-profile in the BLUE-protocol. Compare with the regular lung line of Figure 3. Note the blurred letters due to multiple transfers of this image. Quantitative data: a reasonable thickness at the right image is 5.5 cm, giving an index of 5.5 corresponding to a 165-mL consolidation, roughly. In the left image, the 10-cm depth would correspond to a volume of roughly 1 L. Adapted from “Whole body ultrasonography in the critically ill” (2010 Ed, Chapter 16), with kind permission of Springer Science.

Lung consolidations are fluid disorders and, therefore, are easily traversed by ultrasound. This old potential [2,15,16], long underused in ICUs, benefits from a standardized approach. Lung consolidations touch the wall in 98% of cases [17], arise at any site, making ultrasound sensitivity dependent on the site, size, time spent. Most cases (90%) locate, however, at the PLAPS-point [17]. In the critically ill, consolidations are nontranslobar or translobar, an important distinction because this generates different signs, each quite specific (Figure 4). The sign of nontranslobar consolidation (most cases) is the shred sign: the border between consolidated and aerated lung is irregular, drawing the fractal line, fully opposed to the lung line. The sign of translobar consolidation is the tissue-like sign: it looks like liver. Both signs allow for 90% sensitivity (as explained) and 98% specificity [17]. Other signs are reserved for difficult cases [18]. The dynamic air bronchogram [17] and the lung pulse, which visualizes heart beats at the pleural line through a noninflating lung, can distinguish pneumonia from atelectasis. For quantitative data, see Figure 4.

**Figure 5:**
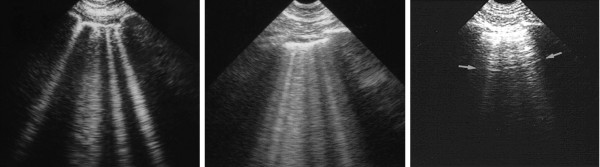
**Interstitial syndrome and the lung rockets.** Two examples of interstitial syndrome. Left: four or five B-lines (see precise description in the text) are visible, called lung rockets (here septal rockets correlating with thickened subpleural interlobular septa). Middle: twice as many B-lines, called ground-glass rockets. Two examples of pulmonary edema (with ground glass areas on CT on the middle figure). Right: Z-lines for comparison. These parasites are ill-defined, short, and do not erase A-lines (arrows), among several criteria. Extract from “Whole body ultrasonography in the critically ill” (2010 Ed, Chapter 17), with kind permission of Springer Science.

Interstitial syndrome is a disorder rarely recognized with usual tools. Intensivists don’t devote much energy to its detection, yet this application has basic, unexpected potential. Our updated definition of the B-line requires three constant and four quite constant criteria [19]. The B-line is always a comet-tail artifact, always arises from the pleural line, and always moves in concert with lung-sliding. It is almost always long, well-defined, laser-like, hyperechoic, erasing A-lines (Figure 5). This definition distinguishes it from all other comet-tail artifacts. Briefly, air and water are simultaneously hit by ultrasound beams, as occurring when subpleural interlobular septa are edematous [20]. Three or more B-lines between two ribs are called lung-rockets. Lung-rockets correlate with interstitial syndrome with 93% accuracy using alveolar-interstitial radiographic changes as reference, and full accuracy using CT [20]. Up to 3–4 B-lines are called septal rockets, correlated with Kerley B-lines [21]. Twice as many, called ground-glass rockets, correlate with ground-glass areas [20]. In the BLUE-protocol, only anterolateral lung-rockets are considered: posterior interstitial changes can be due to gravity alone. Harmonics of modern machines can alter B-lines. The BLUE-protocol can distinguish hemodynamic pulmonary edema from ARDS, COPD, and rule out pneumothorax [22,23] as confirmed [24-27].

**Figure 6:**
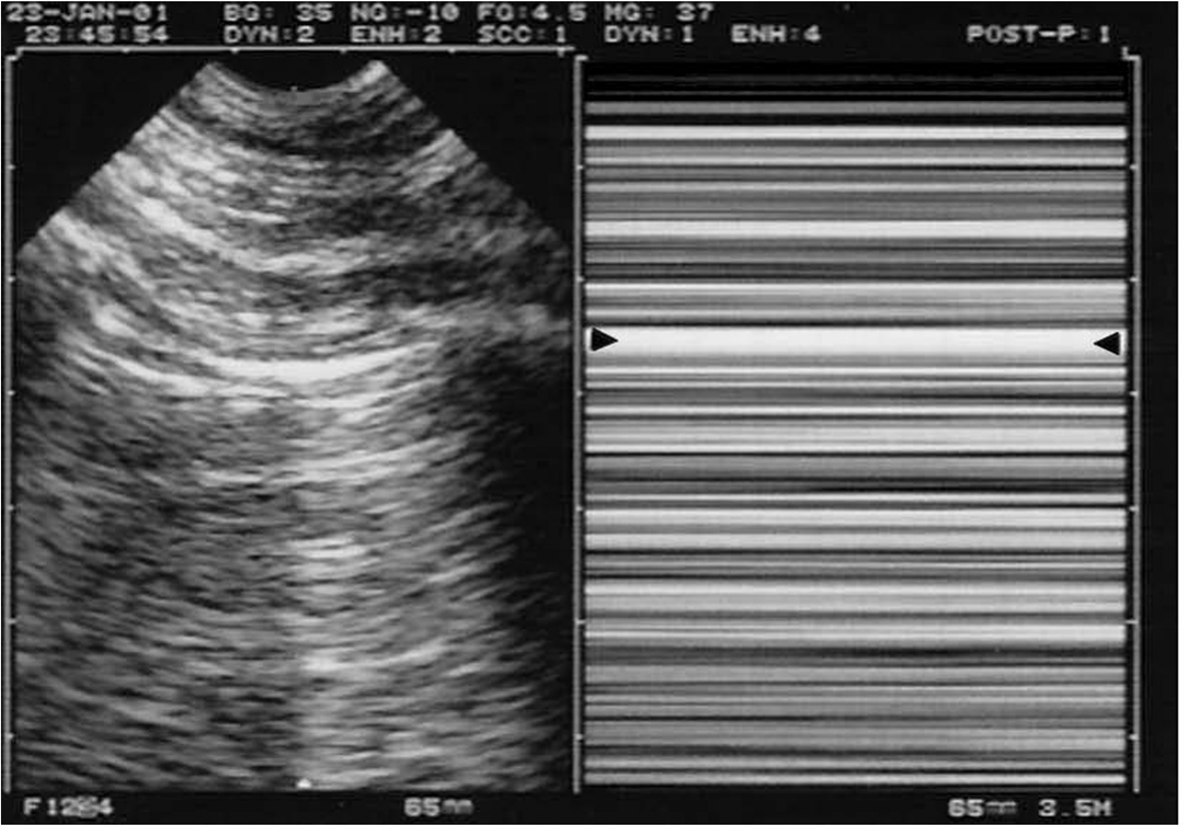
**Pneumothorax and the stratosphere sign.** Left: same pattern as in Figure 2, i.e., pleural line with A-lines, indicating gas below the pleural line. Not visible on the left image, lung sliding is totally absent. Right: here on M-mode, the abolition of lung sliding is visible through the stratosphere sign (which replaces the seashore sign) and indicates total absence of motion. This suggests pneumothorax as a possible cause (see others in text). Arrows: location of the pleural line. The combination of abolished lung sliding with A-lines, at the anterior chest wall, is the A’-profile of the BLUE-protocol (as opposed to the A-profile, where lung sliding is present, ruling out pneumothorax). Extract from “Whole body ultrasonography in the critically ill” (2010 Ed, Chapter 18), with kind permission of Springer Science.

**Figure 7:**
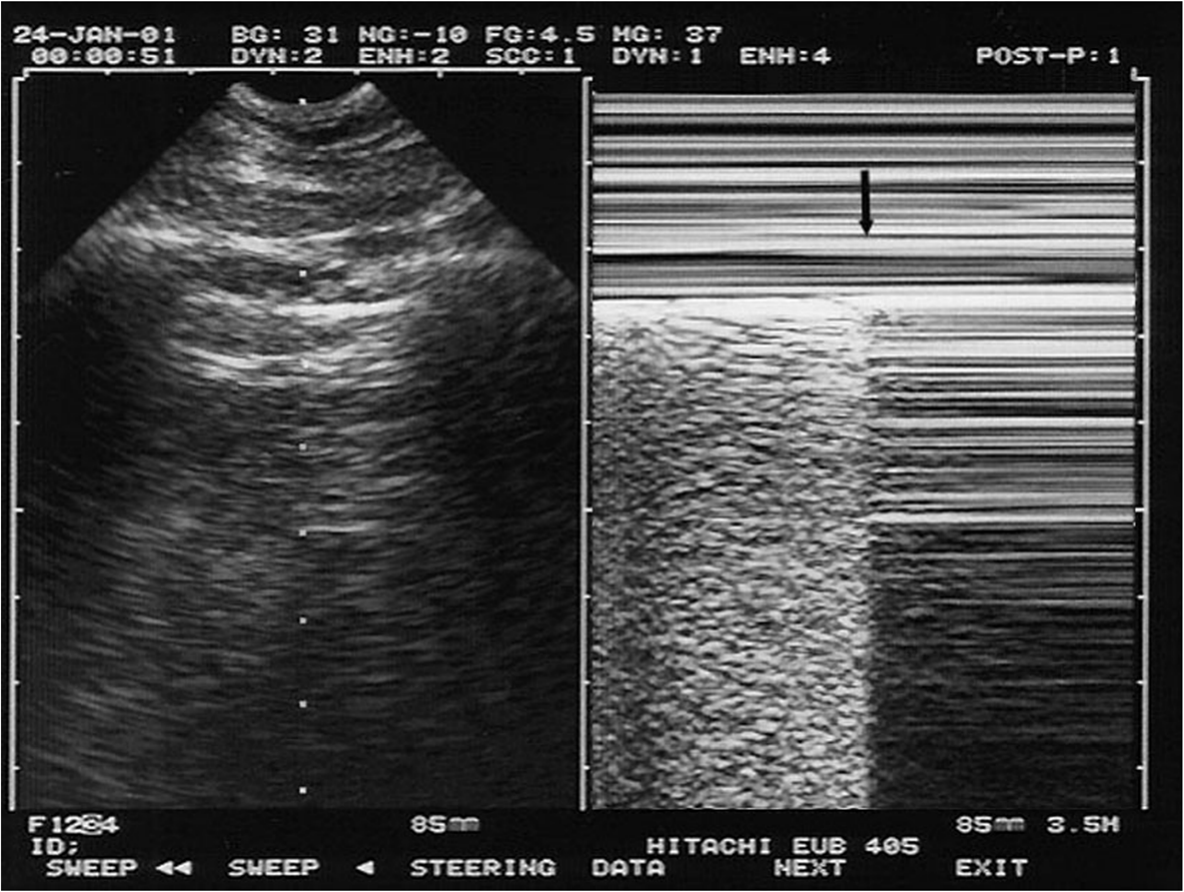
**Pneumothorax and the lung point.** A specific sign of pneumothorax. Real-time mode allows detection of the inspiratory increase in volume of the collapsed lung. When reaching the chest wall where the probe is laid, it makes a sudden change in the ultrasound image, from an A’-profile to an A- or B-profile usually. The change is sudden because (using an appropriate equipment, without average filters or time lag mainly) ultrasound is a highly sensitive method, able to detect subtle changes, such as the difference between free gas and alveolar gas. The left image shows the pleural line just before the visceral pleura appears. The right image shows (arrow) the very moment the visceral pleura has touched the parietal pleural. This sign has been called lung point (it can be seen along a line, but one point is sufficient for the diagnosis). Video visible at CEURF.net. Extract from “Whole body ultrasonography in the critically ill” (2010 Ed, Chapter 18), with kind permission of Springer Science.

Diagnosis of pneumothorax requires three steps. Abolished lung-sliding, long described in horses [28], is found anteriorly in quite all significant cases in supine patients [29]. It has a 95% sensitivity (100% if revisiting methodology) and 100% negative predictive value [30]. Pneumothorax therefore is confidently discounted each time lung-sliding is present, as confirmed [31-34]. Lung-sliding can be extremely moderate, up to the lung-pulse, an equivalent of lung-sliding when searching for pneumothorax. Pneumothorax generates a completely motionless pleural line using real-time. M-mode shows a standardized stratified pattern below and above the pleural line: the stratosphere sign (Figure 6). Dyspnea generates interfering movements above the pleural line. Vascular probes are usually used, but our microconvex probe has no drawbacks, plus the advantage of immediate whole-body assessment. Abolished lung-sliding is everything but specific: inflammatory adherences (i.e., ARDS), atelectasis (one-lung intubation), chronic adherences, fibrosis, phrenic palsy, jet ventilation, cardiopulmonary arrest, apnea, esophageal intubation, inappropriate settings, inappropriate probes are usual factors, and frequent in critically ill patients. The positive predictive value of abolished lung-sliding, only 87% in a general population [30], falls to 56% in the critically ill [35], and to 27% in patients with respiratory failure [36]. The notion of ultrasound “false-positives” makes little sense when another sign is added: the A-line sign (i.e., no B-line seen), with 60% sensitivity but 100% specificity, a logical finding: interlobular septa come only from visceral pleura [23]. One motionless B-line discounts pneumothorax. Too superficial linear probes make it difficult to distinguish B-lines from other comet-tail artifacts (Figure 5). Abolished lung-sliding plus absence of B-lines, at the anterior area, in supine patients, is called A’-profile in the BLUE-protocol (Figure 6). The third step—the lung point—is pathognomonic [35]. It shows in patients with an A’-profile, at a precise location, lung signs suddenly appearing with respiration: transient B-lines, lung-sliding (Figure 7). It is explained by the inspiratory increase of parietal contact of the collapsed lung. Complex pneumothoraces with extensive adherences will not generate any lung-point. The lung-point indicates that abolished lung-sliding is not linked to technical flaws, modern machines, or excessive filters (modern equipments with time lags may generate issues). The sensitivity is 66%: fully collapsed lungs cannot reach the wall. Sensitivity for occult pneumothorax is 79% [37], proving that the lung-point indicates pneumothorax volume: moderate if anterior, massive if posterior or even absent. Lateral lung-points correlate with a 90% need for drainage versus 8% with anterior lung-point [37], as confirmed [34,38]. Some seconds are required for well-trained physicians to determine lung-sliding, B-lines, or their absence—less than 1 minute to detect a lung-point.

The lung-pulse is useful for immediate diagnosis of an atelectasis (one-lung intubation included) [39]. The diaphragm is interesting, but we do not devote much time to careful analysis: locating the thoracoabdominal frontline and its respiratory movement shows where it is and how it works [40].

### Clinical applications of lung ultrasound in the critically ill

How can lung ultrasound become a daily tool for the intensivist? By applying fast protocols devoted to acute respiratory or circulatory failure or cardiac arrest, by limiting irradiation, mainly.

### The approach to acute respiratory failure: the BLUE-protocol

Acute respiratory failure is a life-threatening condition whose cause is sometimes difficult to recognize immediately. Initial mistakes have deleterious consequences [41]. The extreme patient’s suffering legitimizes the use of any tool that expedites relief. Reducing the time needed to provide this relief is the aim of the BLUE-protocol.

The BLUE-protocol, performed on dyspneic patients who will be admitted to the ICU, is a fast protocol: 3 minutes are required using suitable machines and the standardized points of analysis. Novices can take longer (this time depends on the simplicity and adequacy of their equipment, of the standardization of their training). Based on pathophysiology, it provides a step-by-step diagnosis of the main causes of acute respiratory failure, i.e., six diseases seen in 97% of patients in the emergency room, offering an overall 90.5% accuracy [28,42].

**Figure 8:**
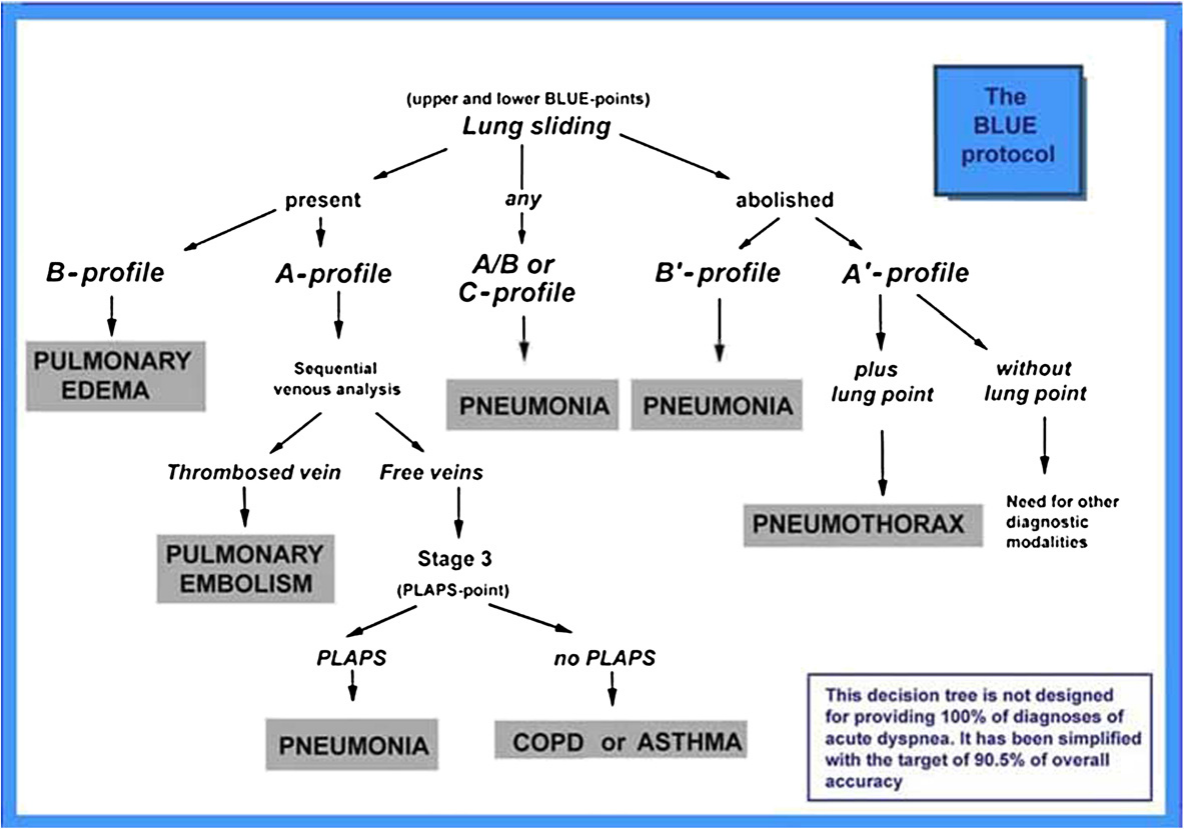
**The BLUE-protocol decision tree.** This decision tree, slightly modified from the original article (Chest 2008;134:117–125), with the permission of *Chest*, indicates a way proposed for immediate diagnosis of the main causes of acute respiratory failure, using a lung and venous ultrasound approach.

The BLUE-protocol combines signs, associates them with a location, resulting in seven profiles (Figure 8).

The A-profile associates anterior lung-sliding with A-lines.

The A’-profile is an A-profile with abolished lung-sliding.

The B-profile associates anterior lung-sliding with lung-rockets.

The B’-profile is a B-profile with abolished lung-sliding.

The C-profile indicates anterior lung consolidation, regardless of size and number. A thickened, irregular pleural line is an equivalent.

The A/B profile is a half A-profile at one lung, a half B-profile at another.

The PLAPS-profile designates PosteroLateral Alveolar and/or Pleural Syndrome. PLAPS are sought for after detection of an A-profile (a pattern compatible with pulmonary embolism) and of a free venous network (a pattern making the diagnosis of embolism less likely). The profile combining A-profile, free veins, and PLAPS is called A-V-PLAPS-profile.

Each profile is associated with a disease, schematically, with accuracy indicated in Table 1.

**Table 1:** Detailed performances of the BLUE-protocol

**Mechanism of dyspnea**	**Profiles of BLUE-protocol**	**Sensitivity**	**Specificity**	**Positive predictive value**	**Negative predictive value**
Acute hemodynamic pulmonary edema	B-profile	97%	95%	87%	99%
		(62/64)	(187/196)	(62/71)	(187/189)
COPD in exacerbation or severe acute asthma	Nude profile	89%	97%	93%	95%
		(74/83)	(172/177)	(74/79)	(172/181)
Pulmonary embolism	A-profile (with deep venous thrombosis)	81%	99%	94%	98%
		(17/21)	(238/239)	(17/18)	(238/242)
Pneumothorax	A’-profile (with lung point)	88%	100%	100%	99%
		(8/9)	(251/251)	(8/8)	(251/252)
Pneumonia	B’-profile	11%	100%	100%	70%
		(9/83)	(177/177)	(9/9)	(177/251)
	A/B profile	14.5%	100%	100%	71.5%
		(12/83)	(177/177)	(12/12)	(177/248)
	C-profile	21.5%	99%	90%	73%
		(18/83)	(175/177)	(18/20)	(175/240)
	A-V-PLAPS profile	42%	96%	83%	78%
		(35/83)	(170/177)	(35/42)	(170/218)
	The four profiles	89%	94%	88%	95%
		(74/83)	(167/177)	(74/84)	(167/176)

Number of patients are shown in parentheses.

The B-profile suggests acute hemodynamic pulmonary edema with 97% sensitivity and 95% specificity. The A-profile associated with DVT provides an 81% sensitivity and 99% specificity for pulmonary embolism. The B’-profile, A/B-profile, C-profile, and A-V-PLAPS profile are typical profiles indicating pneumonia. An A-profile without DVT or PLAPS (the nude profile) is likely to be severe asthma or exacerbated COPD. The A’-profile and a lung-point is specific to pneumothorax.

The BLUE-protocol is initiated just after the physical examination and followed by echocardiography, cardiac windows permitting, restricted to a basic, real-time analysis. Called simple cardiac sonography at CEURF, this approach is increasingly developing [43].

Space lacks to describe many subtleties. Hemodynamic pulmonary edema generates transudate, a kind of oil explaining conserved lung-sliding (B-profile). Pneumonia generates exudate, a kind of glue, explaining the B’-profile. This partly explains the potential for distinguishing ARDS from hemodynamic pulmonary edema. Hemodynamic edema generates the B-profile in 97% of cases; ARDS generates a profile of pneumonia in 86% of cases [36]. This is found again in the Italian literature, under the name of spared areas (A/B-profile), lung consolidations (C-profile), pleural line modifications (C-profile) [44].

Countless subtleties (such as the C’-profile, a C-profile with abolished lung sliding) will be included in the extended BLUE-protocol, a definitive version of the BLUE-protocol, which must be considered as a preliminary approach using simplicity. Auscultation data, echocardiographic data also will be included.

Regarding rare, double, absent causes, read [42]. False-positives and false-negatives are of interest, because ultrasound provided data that questioned a posteriori the value of the “gold standard” [36]. Let us remind that, more than simple CT (which isolated does not have a perfect discriminatory power for a given disease), the “gold standard” was the final diagnosis of the hospitalization report.

### Hemodynamic assessment of circulatory failure using lung ultrasound: FALLS-protocol

Acute circulatory failure is associated with high mortality. Many tools have been successively used [45]. Echocardiography is one of the most popular [1]. This presupposes expertise, suitable cardiac windows, or transesophageal approach. Here, we use a fast protocol again based on pathophysiology. The heart approach is limited to the simple cardiac sonography. The lung approach will compensate for any lack of echocardiographic expertise, considering a direct parameter of clinical volemia.

Data for using the FALLS-protocol (Fluid Administration Limited by Lung Sonography) have been published, showing the correlation between an A-profile or equivalents (A/B-profile) and a low pulmonary artery occlusion pressure (PAOP), with a 18-mmHg value occurring when B-lines appear [46]. Caval vein analysis is associated to the FALLS-protocol, especially in the case of initial B-profile.

The FALLS-protocol follows Weil’s classification of shock. It first searches for substantial pericardial effusion (likened to pericardial tamponade in acute circulatory failure), then for right ventricle dilatation (suggesting, in this context, pulmonary embolism, schematically). If the cardiac windows are suboptimal, the BLUE-protocol is used instead. Then, tension pneumothorax is sought for. If these disorders are absent, obstructive shock can be discounted, schematically.

Cardiogenic shock from the left heart (i.e., most cases) is defined by low cardiac output and high PAOP. In the absence of a B-profile, such cardiogenic shock can be discounted.

The remaining causes are hypovolemic and distributive shock. At this step, patients with the A-profile or equivalents, proving dry lungs, are called FALLS-responders. They are those who can, but mostly must, receive fluids, a therapy common to both causes. The FALLS-protocol per se begins: fluid administration.

A hypovolemic mechanism will benefit from fluid therapy, with corrections of the circulatory failure, and unchanged A-profile.

If no clinical improvement occurs, fluids eventually penetrate the lung, which is normally fluid-free. Interstitial edema always precedes alveolar edema [47] and is detected by ultrasound at an early step clinically silent, before gas exchange impairment [48,49]. The change from A- to B-lines indicates the endpoint for fluid therapy. Associated with no improvement of circulatory failure, this indicates, schematically, the only remaining mechanism: distributive shock, meaning in current practice septic shock (obvious diagnoses such as anaphylactic shock or rarities being excluded). This septic shock has just benefited from one major therapy, following the current guidelines [50], with two advantages. Early fluid therapy in sepsis? Far before the diagnosis of septic shock. Massive? Up to the last admissible drop using pathophysiological basis. The intensivist can now consider that this fluid therapy, generating interstitial edema (even silent), has positioned the heart at the beginning of the flat portion of the Frank-Starling curve. Minute fluid withdrawal is achieved, from hemodiafiltration if already present, reversion of passive leg raising (“FALLS-PLR”-protocol), to simple blood cultures, specifically useful here, with a view to positioning the heart at the ideal point of the curve.

If a B-profile is seen on admission, the FALLS-protocol cannot be used. The diagnosis is usually cardiogenic shock, but sometimes lung sepsis. The inferior caval vein roughly correlates with volemia [51,52]. The superior caval vein is accessible to our microconvex probe. Small dimensions, inspiratory collapse suggest hypovolemia [53].

Questions are answered in [54]. One cannot pretend that the FALLS-protocol answers such a complex field; it is open to any criticism. A validation should raise the issue of the choice of a pertinent “gold standard.” Physicians can surround the FALLS-protocol with traditional tools. The change from A-lines to B-lines, which defines septic shock in the FALLS-protocol, can be considered as a direct marker of clinical volemia. Schematically, A-lines indicate fluid responders, B-lines an endpoint for fluid therapy, making FALLS-protocol not comparable to approaches assessing cardiac output. It provides a parameter independent of usual limitations (transmural pressures, cardiac arrhythmia, invasive procedures, etc.). One point should be understood: the caval vein is usually analyzed for predicting fluid responsiveness: fluid is given, cardiac output monitored. FALLS-protocol does not search for any cardiac output increase. In the described sequence, the A-profile indicates that fluid can (and must) be administered. The B-profile on admission (or appearing during fluid therapy) indicates that the patient is (or becomes) an equivalent of not fluid-responder. FALLS-protocol provides a static parameter, which therefore can be used at the start (unlike dynamic parameters).

### Cardiac arrest: the opportunity for technical considerations

Ultrasound plays a major role when showing reversible causes. The SESAME-protocol, a fast protocol devoted to cardiac arrest, assesses the lung before the heart, because pneumothorax can be discounted in 2 seconds, with in addition, windows usually available. This apparently futile property influences the choice of equipment. The following section is personal and subjective. A valuable combination may be our kind of equipment, coupled with high-level Echo machines used every time needed, as we repeatedly wrote [55].

Nowadays machines are good. Each probe is good for its devoted application (vascular, cardiac, abdominal). We just advocate to have, before the current trend, defined critical ultrasound using (after the perfectly suitable ADR-4000) a unit built from 1992 to 2010 which was not inferior, especially in the specific setting of cardiac arrest, and made every step more simple [6]. This machine that we now use every day is 30-cm wide on the cart (no matter its height), i.e., narrower than most machines, laptops with carts included. This answered to the problem of the economy of room in busy ICUs, ORs, ERs, where each saved centimeter makes a difference. It starts in 7 seconds, a critical point in cardiac arrest (in machines with longer start-up, there is nothing to do but wait). Its microconvex probe is a compromise allowing in a few seconds, lungs, heart, vessels, abdomen assessment exploiting its 17-cm range, revealing reversible causes (pneumothorax, tamponade, venous thrombosis, abdominal bleeding…). It is flat, therefore cleanable, keyboard highlights three basic knobs useful in extreme emergencies: gain, depth, M-mode. Its technology does not filter out the artifacts and does not create time lags. Its low cost was an opportunity for most patients on Earth. Each detail interacts with the others, e.g., our single probe lies on our machine top, not laterally, a detail that saves lateral width. Our main work was to optimize each step. Our slim machine is permanently configurated “cardiac arrest,” which works the same, without necessary change, for routine, daily tasks (venous line insertion…). Some manufacturers begin to build machines inspired by this 1992 technology.

Unexpected limitations (dealt with in our textbook, some apparently futile) can suddenly appear at any step of the management of extreme emergencies, potentialized by the extreme stress. An issue is the permanent risk to face unsuitable cardiac windows. If the user wishes to follow the SESAME-protocol, i.e., assessing here the veins (especially calf areas), the cardiac probe should be urgently replaced by a vascular probe. Time is necessary at each probe change (heart, abdomen, lungs…), setting change, not to forget probe/cable disinfection (here theoretical, usually a critical point). Complex keyboards turn into hindrances to novices. Several probes make cables inextricably mixed. Cables lying on the floor favor the risk of a machine tipover when suddenly mobilized. Problems occur when each of these small difficulties is added to each other.

For expediting the mastery of lung ultrasound, we advise to bypass all filters (a setting one may call “lung”). Each probe provides fractional data (abdominal probe for pleural-alveolar characterization, cardiac probe for posterior analysis in challenging patients, vascular probe if others cannot show lung-sliding, abdominal again for assessment of artifacts length, etc.). Most microconvex probes found in laptop machines do not have the resolution or range of ours. Machines with lag between real-time and M-mode can confuse young or stressed users. Physicians also should check that their cardiac probes are able to document lung sliding in all conditions (skinny patients, dyspnea, etc.).

This section was an opportunity to emphasize the interest of our universal probe among others [56]. We think each user, even expert, should try similar systems, at least once.

## Lung ultrasound: a holistic discipline

A perspective is holistic when the relevance of each of its multiple element can be understood only if integrated with the others. Lung ultrasound makes ultrasound a holistic discipline, as partially seen in the previous section.

### Multifaceted tool

Lung ultrasound can be used without complex adaptation from the intensivist to anesthesiologists, pediatricians, neonatal intensivists, emergency physicians, and others (cardiologists, pulmonologists, nephrologists, etc.), even out-of-hospital doctors [57]. The lung is a common target in these disciplines. The signs assessed using CT in adults are found without difference in critically ill neonates [58,59]. The unit is easily affordable, generating huge cost-cutting [39]. These potentials are applicable from sophisticated ICUs to more basic settings on Earth. Lung ultrasound complements poor cardiac windows: B-profile shows pulmonary edema, A-profile hypovolemia, schematically. Its feasibility is nearly 100%: this vital organ is superficial and extensive, including bariatric patients, where the anterior approach provides basic data. Painful blood gas analyses become less relevant.

### Attractive tool

Lung ultrasound is not really ultrasound (i.e., this expert, operator-dependent tool) for several reasons. Just two signs are sufficient to define the normality (lung-sliding, A-lines). This potential allows us to reconsider usual priorities. Once the physicians operational for life-saving protocols (BLUE-protocol, FALLS-protocol), they can quietly learn comprehensive echocardiography during as long time as necessary.

### Solution to the issue of growing irradiation

All intensivists prefer the least invasive tool, all else being equal. Ultrasound is an answer to the longstanding dilemma: “Radiography or CT in the ICU?” Radiography is a familiar tool that lacks sensitivity [60]: 60-70%, all fields considered [61-63]. CT has a high accuracy but severe drawbacks: cost (a real problem for most patients on Earth), transportation of critically ill patients, delay between CT and the resulting therapy, renal issues, anaphylactic shock, mainly high irradiation [64,65]. Ultrasound has quite similar performances to CT [12,17,20,30,37], being on occasion superior: better detection of pleural septations, necrotic areas [66], real-time measurement allowing assessment of dynamic signs: lung-sliding, air bronchogram [67], diaphragm [68,69]. Ultrasound should be considered as reasonable, bedside “gold standard.” For all assessed disorders, it provides quantitative data (Figures 3, 4, and 7). Pleural effusions can be quantified [14,70-72]. Lung consolidation can be monitored, which is useful for those who want to increase end-expiratory pressure [73]. The volume and progression of a pneumothorax are monitored using the lung-point location [34,37,38]. Lung ultrasound will favor programs allowing decrease in bedside radiographs and CTs in the next decades.

### Limitations

Dressings and subcutaneous emphysema make unsuperable limitations. Exceptional cases provide difficult interpretation, even for experts. Is lung ultrasound easy? Some experiences show high interobserver agreement [13]. A burgeoning literature, up to a consensus conference [74-88], seems to confirm this accessibility. A scientific assessment of the learning curve remains to be done, not in volunteers (creating a selection bias), but in unselected physicians. Care should be taken to confide training to experts choosing simplicity, although one can practice lung ultrasound with any machine, any probe, any teaching approach. Our work was mainly to provide standardized signs, a major advantage of lung ultrasound, because the risk of wrong interpretations is highly decreased.

## Review, conclusions

Lung ultrasound allows fast, accurate, bedside examinations of most acute respiratory disorders. It enables a pathophysiological approach to circulatory failure. Simplicity is providentially found at this vital organ. The versatility of lung ultrasound heralds a kind of visual medicine, a priority in intensive care as well as many other disciplines and settings [89].

### Videos

Videos are available at http://www.CEURF.net, section BLUE-protocol.

## Competing interest

The author declares that he has no competing interests.
